# Biomaterial-Supported Cell Transplantation Treatments for Spinal Cord Injury: Challenges and Perspectives

**DOI:** 10.3389/fncel.2017.00430

**Published:** 2018-01-11

**Authors:** Shengwen Liu, Thomas Schackel, Norbert Weidner, Radhika Puttagunta

**Affiliations:** ^1^Spinal Cord Injury Center, Heidelberg University Hospital, Heidelberg, Germany; ^2^Department of Neurosurgery, Tongji Hospital, Tongji Medical College, Huazhong University of Science and Technology, Wuhan, China

**Keywords:** cell transplantation, biomaterial scaffolds, spinal cord injury, axonal regeneration, combinatorial therapy

## Abstract

Spinal cord injury (SCI), resulting in para- and tetraplegia caused by the partial or complete disruption of descending motor and ascending sensory neurons, represents a complex neurological condition that remains incurable. Following SCI, numerous obstacles comprising of the loss of neural tissue (neurons, astrocytes, and oligodendrocytes), formation of a cavity, inflammation, loss of neuronal circuitry and function must be overcome. Given the multifaceted primary and secondary injury events that occur with SCI treatment options are likely to require combinatorial therapies. While several methods have been explored, only the intersection of two, cell transplantation and biomaterial implantation, will be addressed in detail here. Owing to the constant advance of cell culture technologies, cell-based transplantation has come to the forefront of SCI treatment in order to replace/protect damaged tissue and provide physical as well as trophic support for axonal regrowth. Biomaterial scaffolds provide cells with a protected environment from the surrounding lesion, in addition to bridging extensive damage and providing physical and directional support for axonal regrowth. Moreover, in this combinatorial approach cell transplantation improves scaffold integration and therefore regenerative growth potential. Here, we review the advances in combinatorial therapies of Schwann cells (SCs), astrocytes, olfactory ensheathing cells (OECs), mesenchymal stem cells, as well as neural stem and progenitor cells (NSPCs) with various biomaterial scaffolds.

## Introduction

Traumatic spinal cord injury (SCI) results in the disruption of neuronal circuitry leading to the partial or complete loss of motor control, autonomic function and sensory input. Paraplegic or tetraplegic patients must also contend with chronic consequences ranging from spasticity, neuropathic pain, bladder and bowel dysfunction, pressure ulcers, respiratory and cardiovascular complications which significantly decreases quality of life. World-wide SCI has an annual incidence ranging from 20 to 30 patients per million people (Lee et al., 2014; Singh et al., 2014; Jain et al., 2015; Jazayeri et al., 2015). However, to date there is no effective SCI therapy that can entirely restore neurological deficits. Such therapies must address various complex obstacles that develop after SCI, in particular, cyst formation, neural cell death, a growth-inhibitory microenvironment, scar formation, demyelination, and the disruption of the blood supply (Silva et al., 2014). Although, any treatment option that allows a patient to partially regain lost neuronal circuitry, whether it be motor, sensory or autonomic, will prove to be invaluably beneficial.

In previous decades, a myriad of experimental studies have been conducted to develop potential treatment options for SCI patients. Many studies showed a certain degree of morphological changes partially accompanied by behavioral improvements in various animal models (Fouad et al., 2005; Blesch and Tuszynski, 2009; Franz et al., 2012; McCall et al., 2012; Zhao et al., 2013; Danilov and Steward, 2015; Gomes-Osman et al., 2016; Kadoya et al., 2016). Amongst these studies, cell-based transplantation has been considered as a promising therapeutic strategy due to: (1) direct replacement of the damaged neural tissue, (2) neuroprotective properties for spared neuronal connections, and (3) providing a permissive and supportive cellular growth substrate for axonal regrowth and/or plasticity (Ohta et al., 2004; Feron et al., 2005; Granger et al., 2014; Kanno et al., 2015). Obstacles for cell-based transplantation therapy remain to be the low survival rates of the grafted cells after transplantation into the injured spinal cord, retention of grafted cell at the lesion site without migration, filling the lesion cavity that has formed as well as directional guidance of axonal growth (Pearse and Barakat, 2006; Pearse et al., 2007; Parr et al., 2008; Takahashi et al., 2011). Although a huge effort has been made to modify delivery mechanisms and surgical techniques, success has been modest and relatively inconsistent. In addition to the beneficial effects stated above, some drawbacks of cell transplantation must also be stated and further explored, including tumorgenic formation (Matsuda et al., 2009; Fu et al., 2012; Liu et al., 2013), maladaptive plasticity such as pain hypersensibility (Hofstetter et al., 2005; Macias et al., 2006; Davies et al., 2008), non-beneficial differentiation or dedifferentiation (Hill et al., 2004; Lepore et al., 2004), increased immunoreactivity to transplanted cells (Swanger et al., 2005), complications arising from surgical delivery (Takahashi et al., 2011) and deficits due to immunosuppression (Antonic et al., 2013). Nonetheless, some candidate cell types have already been investigated in clinical trials in SCI patients such as autologous (cells from the same individual) Schwann cells (SCs) (Anderson et al., 2017), olfactory ensheathing cells (OECs) (Mackay-Sim et al., 2008; Tabakow et al., 2013), bone-marrow mesenchymal stem cells (BMSCs) (Park et al., 2005; Kumar et al., 2009; Karamouzian et al., 2012; Mendonca et al., 2014; Oraee-Yazdani et al., 2016) and different neural stem and progenitor cells (NSPCs) (Shin et al., 2015).

Biomaterials have been combined with various cell types to address the issues of cell viability, cell retention at the lesion site, supportive physical matrix, filling of the lesion cavity as well as mediating directed growth (Atala, 2000; Madigan et al., 2009; Cao et al., 2011; Luo et al., 2016; Ogle et al., 2016). Numerous studies have proven diverse biomaterials to be appropriate delivery vehicles for cells as well as bioactive molecules and drugs in different injury and disease models in the central nervous system (CNS) (Krishna et al., 2013; Führmann et al., 2017).

## Function of cell-seeded biomaterials in experimental sci models

Biomaterial scaffolds can fulfill multiple functions for SCI transplantation approaches: (1) specific three-dimensional microarchitectures can be designed with small “chambers” or aligned channels/fibers suited for cell seeding and axonal growth in a directed linear pattern facilitating substantial axonal growth across the lesion for establishment of synaptic connections (Gros et al., 2010; Gunther et al., 2015a; Onuma-Ukegawa et al., 2015); (2) serves as a physical matrix for cell adhesion and thereby enhancing survival and retention of grafted cells at the lesion site (Hurtado et al., 2006; Olson et al., 2009; Bozkurt et al., 2010; Park et al., 2012) and affect host cell migration (e.g., SCs and astrocytes) (Suzuki et al., 2015); (3) influence the behavior of grafted cells and differentiation (Mekhail et al., 2015); and (4) control the release of encapsulated bio-active molecules (Mothe et al., 2013).

Biomaterials can be fabricated from natural or synthetic polymers and subdivided into three major forms: solid scaffolds, hydrogels and micro-/nanoparticles (Boisserand et al., 2016). Various types of biomaterials have been explored in tissue engineering for SCI repair (Table 1). Crosslinking of hydrogels typically increases the overall long-term stability of biomaterials, however this also increases the stiffness and the balance between stiffness and stability is a delicate one for cell adhesion, migration and neuroregenerative work (Khaing et al., 2011; Seyedhassantehrani et al., 2016). Additionally, surface modification with extracellular matrix (ECM) components, e.g., laminin and fibronectin, or synthetic peptides represents another way to improve cell adhesion and survival by generating a less hostile molecular microenvironment within the biomaterial (Miller et al., 2001; Chen et al., 2009). Injectable *in situ* polymerizing hydrogels help to deliver cells and factors directly into a lesion site with less invasive surgical interventions, forming a homogenous three-dimensional matrix mimicking natural ECM microstructure to modulate cell fate (Bidarra et al., 2014; Führmann et al., 2016). Importantly, biomaterials can effectively fill a cystic cavity and bridge the lesion dramatically reducing the number of cells required for transplantation. This is particularly appealing for clinical use since the availability of autologous cells from patients is limited.

**Table 1:** Biomaterials of different origins used for animal SCI experimentation.

**Origin**	**Biomaterials**
Natural	Agarose
	Alginate
	Chitosan
	Collagen
	Fibrin
	Fibronectin
	Gellan gum
	Hyaluronan
	Hyaluronic acid
Synthetic	Calcium sulfate cement
	Oligo[poly(ethylene glycol) fumarate] (OPF)
	Poly(ethylene glycol) (PEG)
	Poly-b-hydroxybutyrate (PHB)
	Poly(2-hydroxyethylmethacrylate) (PHEMA)
	Poly(D, L-lactic acid) (PLA)
	Poly(lactide-co-glycolide) (PLG)
	Poly(lactic-co-glycolic acid) (PLGA)

**Figure 1:**
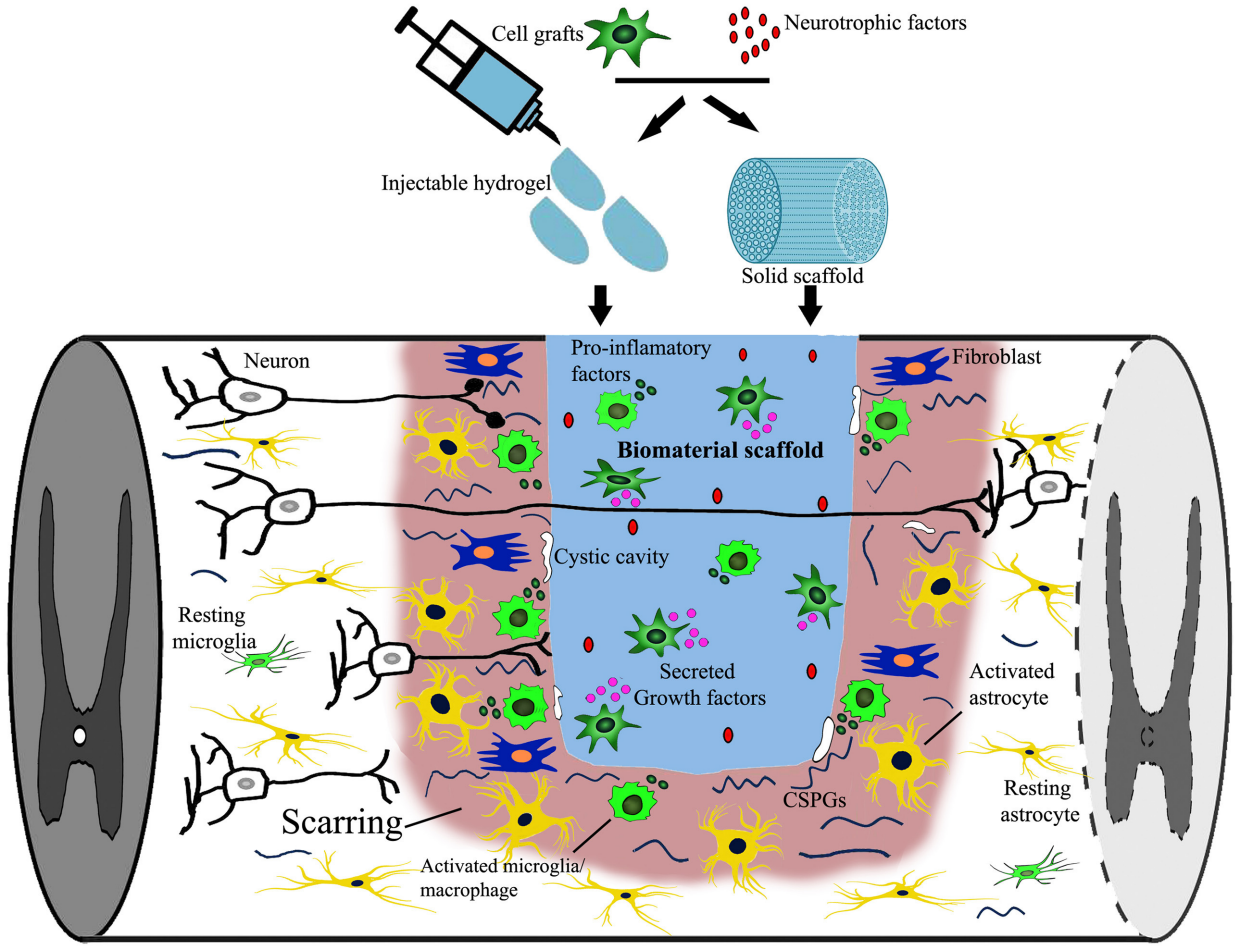
Targets of combined biomaterial-supported cell transplantation following SCI. SCI induced neural damage leads to severed connections, tissue loss and the appearance of a cystic cavity. Combined biomaterials and cell transplantation can be used to fill the lesion cavity to provide physical support and bridge the distance that regrowing axons must traverse. Cell transplantation as well as newly introduced neurotrophic factors may provide trophic support (secreted growth factors) supporting axonal growth. An immune reaction, consisting of activated microglia, macrophages, fibroblasts and astrocytes, is elicited following injury to close off and prevent the spread of damage as well as re-establish the blood-spinal-barrier, possibly obstructing axonal regrowth. Additionally, neural injury leads to the release of growth inhibitory components [myelin debris, inflammatory cytokines, and chondroitin sulfate proteoglycans (CSPG)] that can be down regulated by either cell transplantation or the release of biomolecules into the lesion site.

The potential of biomaterial application alone in SCI treatment has been explored in numerous pre-clinical studies and now even clinical trials (Carballo-Molina and Velasco, 2015; Siebert et al., 2015; Theodore et al., 2016; Xiao et al., 2016). Fibroglial scarring around the graft is a prominent phenomenom after biomaterial scaffold implantation. Typically, only sparse axons re-enter the caudal host spinal cord, while most are confined within the scar surrounding the implants (Suzuki et al., 2002; Grulova et al., 2015; Gunther et al., 2015b; Pawar et al., 2015a; Figure 1). To overcome this barrier without interference with its beneficial roles, targeting inhibitory molecules is one possible solution. For instance, delivery of Chondroitin sulfate proteoglycan (CSPG) cleaving enzyme chondroitinase ABC (ChABC) rostral and caudal to the graft was able to facilitate axonal growth through and beyond the scar (Fouad et al., 2005). Alternatively, cell injections into the host parenchyma around the biomaterial implantation site provide a continous permissive cellular substrate spanning the lesion cavity and biomaterial bridge (tissue bridging) (Ramon-Cueto et al., 1998; Fouad et al., 2005; Deumens et al., 2006a; Liu et al., 2017).

In this review, we assess combinatorial strategies of biomaterial-supported cell transplantation to reconstruct lost host tissue physically, cellularly and chemically after SCI. This includes the integration of biomaterials into the host tissue, bridging the host-graft interface, limiting the effect of the surrounding scar formation which may prevent axonal growth into and through the injury site as well as increasing cell survival to provide the axons with physical, directional guidance and trophic support to regenerate toward disconnected targets (Geller and Fawcett, 2002; Tetzlaff et al., 2011; Kim et al., 2014; Assuncao-Silva et al., 2015; Wu et al., 2015; Lin et al., 2016). Candidate cell populations that enhance biomaterial integration into host tissue such as SCs, astrocytes, OECs, mesenchymal stem cells as well as NSPCs (Tetzlaff et al., 2011; Wu et al., 2015; Badner et al., 2017) will be discussed here.

**Figure 2:**
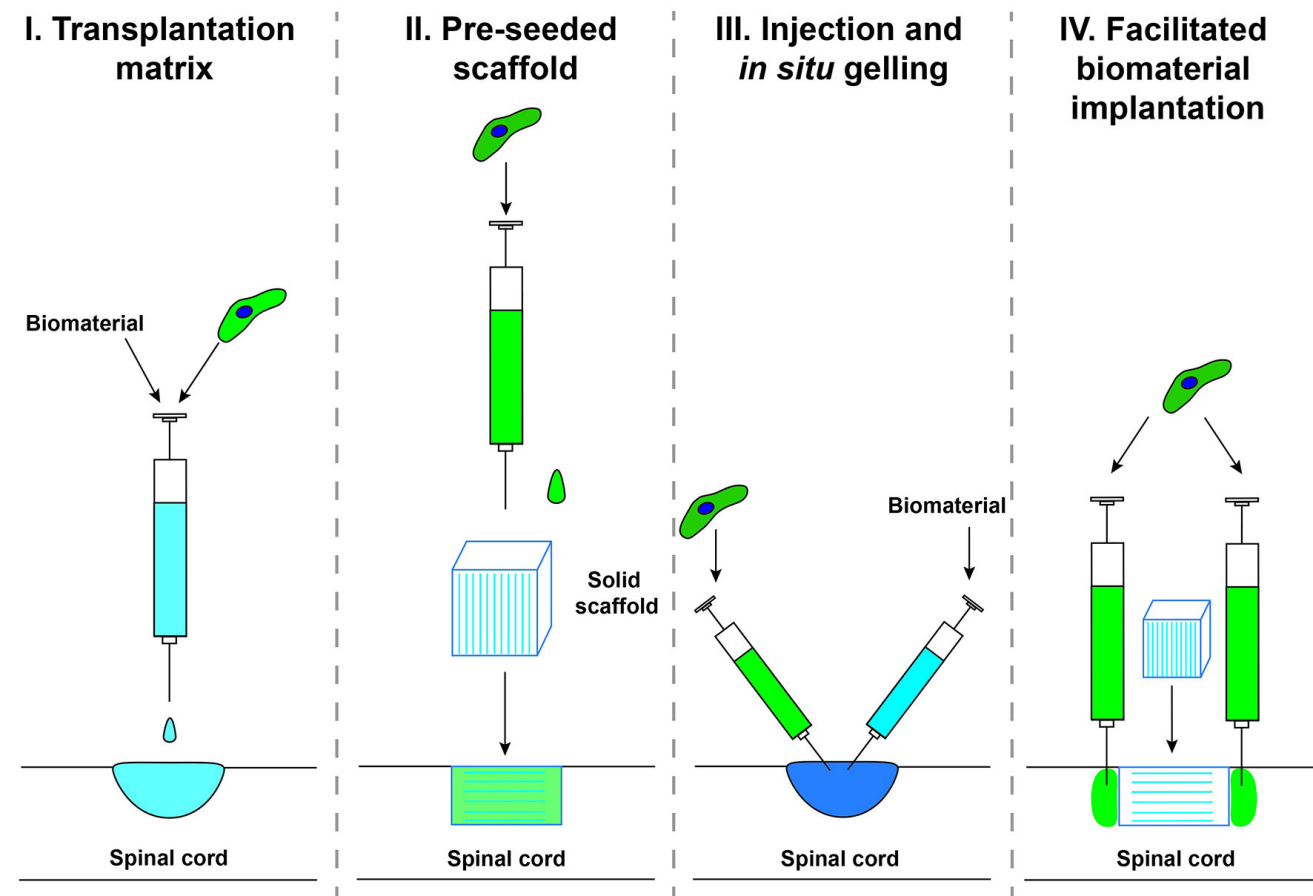
Delivery methods for biomaterials combined with cell transplantation.

The delivery method of biomaterials and cells into a SCI has been undertaken by several different techniques which we will group into categories here for reference throughout the review (Figure 2). Category I, transplantation matrix, is when cells and biomaterials are mixed together *in vitro* and allowed to form a matrix prior to implantation. This technique has been widely used as a delivery system to confine the transplanted cells to the injury site and will not be covered extensively in this review. Category II, pre-seeded scaffold, is when a pre-fabricated biomaterial is seeded with cells prior to implantation. This technique is primarily used for solid scaffolds with a pre-determined shape. Category III, injection and *in situ* gelling, is when self-assembling biomaterials are injected along with cells into the injury site to assemble a seeded scaffold *in vivo*. This technique has become popular to fill irregular lesion cavities that form after SCI. Category IV, facilitated biomaterial implantation, is when a biomaterial is implanted and cells are injected surrounding the biomaterial. This technique has been used to increase the integration of the scaffold into the host tissue, to increase axonal bridging.

## Cellular candidates

### Schwann cells

In the peripheral nervous system, SCs are vital for regenerative growth following an axonal injury by providing trophic and physical support as well as remyelination (Rath et al., 1995; Triolo et al., 2006). SCs secrete ECM components (Tabesh et al., 2009) and neurotrophic factors such as brain-derived neurotrophic factor (BDNF), neurotrophin-3 (NT-3), nerve growth factor (NGF) (Dey et al., 2013; Godinho et al., 2013) and glial cell-derived neurotrophic factor (GDNF) (Iannotti et al., 2004). Therefore, transplantation of SCs after SCI has been widely studied and numerous results demonstrate that SCs can support survival and maintenance of spared neural tissue, bridge lesion cavities, promote re-growth of both motor and sensory axons into the lesion after engraftment and remyelinate CNS axons (Xu et al., 1997; Weidner et al., 1999; Marcol et al., 2015). Unfortunately, SCs form a distinct divide between themselves and CNS tissue both *in vitro* and *in vivo* (Ghirnikar and Eng, 1994; Lakatos et al., 2000). A reformation of the glial limitans and increased production of growth inhibitory CSPG (Plant et al., 2001) likely restrict the regenerative effect of SCs on descending motor neuronal tracts (Vroemen et al., 2007; Kanno et al., 2014).

Xu and colleagues conducted a series of studies demonstrating that naïve SCs or SCs overexpressing neurotropic factors embedded in a semi-permeable single channel composed of polyacrylonitrile and polyvinylchloride copolymers (PAN/PVC) (Category II) in T8 hemisection and transection rat SCI models enhanced the growth of propriospinal and some supraspinal axons into the lesion (Xu et al., 1995a,b, 1997, 1999). However, most often axons did not exit the lesion site on the caudal side likely due to the formation of the glial limitans restricting the SC migration and further beneficial effects. In addition, in a rat C4 2–3 mm hemisection model, biodegradable tubular poly-β-hydroxybutyrate (PHB) scaffolds filled with SCs (Category II) were able to support the survival of the SCs by promoting attachment as well as facilitating raphespinal and sensory axonal growth within the conduit; similar to previous observations, no rubrospinal or corticospinal tract (CST) re-growth was observed (Novikova et al., 2008).

To address the lack of re-innervation of the uninjured host parenchyma caudal to the biomaterial bridge by regenerating axons one aspect is to limit the formation of the glial limitans and reactive astrogliosis. One method that at least extended growth of descending axons (serotonergic) back out of a 2 mm alginate-based anisotropic capillary hydrogel in a C4 unilateral hemisection was the injection of SCs caudal to the SC-seeded hydrogel with the additional caudal viral expression of BDNF (Liu et al., 2017) (Category II and IV). Further work needs to be done to elucidate if this moved the glial limitans further down the cord to the host spinal injection site of SCs or if growth past the grafted SCs is possible. It was found in a 4 mm rat T8 complete transection that the unique combination of SC in fluid Matrigel in a PAN/PVC single channel scaffold, with OEC grafting in host parenchyma surrounding the lesion (Category II, III, and IV) and the delivery of ChABC led to functional improvement (BBB motor recovery score; up to 6 at 9 weeks vs. 2 with no treatment) (Fouad et al., 2005). Although this did not correlate to increased serotonergic fiber number caudal to the injury site but to SC myelinated fibers in the lesion site. Another approach to limit reactive astrogliosis (reduction of GFAP upregulation and CSPG expression) as well as further the intermingling of SCs and astrocytes within the graft and host tissue was the genetic modification of SCs to overexpress GDNF in a single PAN/PVC channel in a 3 mm T9-T10 hemisection rat model which led to enhanced SC remyelination of regenerating axons that were also aligned with protruding astrocytic extensions (Category II) (Deng et al., 2011). Additionally, SC grafting without neurotrophic factor delivery in a full T8 transection with fluid Matrigel (Category III) vs. pre-gelled Matrigel (Category I) in a PAN/PVC single channel (combined with Category II) showed greater growth of virally traced brainstem-derived (vestibular, noradrenergic, serotonergic, and reticular nuclei) axons into and up to the caudal interface where they are believed to have formed synaptic junctions with the help of newly formed supportive astrocytic protrusions (Williams et al., 2015). This significantly correlated to increased motor recovery as scored by BBB. It was proposed that fluid Matrigel led to better bridging of the host-graft interface and limited meningeal cell infiltration restricting the re-establishment of the glial limitans.

SC transplantation alone has been found to be beneficial, however the combination of SCs with biomaterials and other components provided substantial enhanced axonal regrowth and thereby functional improvement by eliciting better host-graft integration (Table 2). This includes the migration of SCs into the host parenchyma as well as astrocytic protrusions into the graft, the reduction of the glial limitans and reactive astrogliosis.

**Table 2:** Schwann Cell-seeded biomaterial SCI studies.

**SCI model**	**Biomaterial**	**Cell type**	**Outcome**	**References**
Rat T8 transection	5 mm PAN/PVC and Matrigel (Category II)	Adult rat SC + BDNF and NT-3 infusion	Increased axonal growth up to biomaterial with NTFs	Xu et al., 1995a
Rat T8 transection	10 mm PAN/PVC and Matrigel (Category II)	Adult rat SC	Increased myelination of propriospinal and sensory axons within the scaffold but not exiting	Xu et al., 1995b
Rat T8 transection	8 mm PAN/PVC and Matrigel (Category II)	Adult rat SC	Increased myelination of propriospinal and sensory axons within the scaffold but not exiting	Xu et al., 1997
Rat T8 hemisection	5 mm PAN/PVC and Matrigel (Category II)	Adult rat SC	Increased myelination of propriospinal and sensory axons within the scaffold with some exiting	Xu et al., 1999
Rat C4 hemisection	2–3 mm PHB (Category II)	Adult rat SC	Some raphespinal and sensory axonal growth within the scaffold	Novikova et al., 2008
Rat C4 unilateral hemisection	2 mm alginate-based anisotropic capillary hydrogel (Category II and IV)	Adult rat GFP-SC	Serotonergic growth through and caudal to the biomaterial up to the 1 mm SC injection site	Liu et al., 2017
Rat T8 transection	4 mm PAN/PVC and fluid or pre-gelled Matrigel (Category I, II, III and IV)	Adult rat SC and OEC surrounding the lesion + ChABC	Increased SC myelination within the scaffold with increased BBB scores	Fouad et al., 2005
Rat T9-T10 hemisection	3 mm PAN/PVC (Categroy II)	Adult rat SC expressing GDNF	Enhanced SC remyelination of regenerating axons aligned with protruding astrocytic extensions	Deng et al., 2011
Rat T8 transection	4 mm PAN/PVC and fluid or pre-gelled Matrigel (Category I, II, and III)	Adult rat SC	Greater growth of virally traced brainstem-derived axons into and up to the caudal interface, formed synaptic junctions with the help of newly formed supportive astrocytic protrusions	Williams et al., 2015

### Astrocytes/glial-restricted progenitors/glial-restricted progenitor-derived astrocytes

Making up the majority of the glia cells in the CNS, astrocytes fulfill essential homeostatic and supportive functions such as providing an organized physical matrix as well as producing neurotrophic factors for axonal remodeling and plasticity (Powell and Geller, 1999; Kimelberg and Nedergaard, 2010; Lukovic et al., 2015). In response to SCI, a dense network of reactive astrocytes physically restricts the lesion site by preventing the spread of neuroinflammation and necrosis immediately after injury (Faulkner et al., 2004; Sofroniew and Vinters, 2010; Anderson et al., 2016), although its persistence in the chronic phase may pose a physical and chemical barrier for re-growing axons (Smith-Thomas et al., 1994; Afshari et al., 2009; Sofroniew, 2009; Hellal et al., 2011; Cregg et al., 2014). Nonetheless, reactive astrogliosis is an essential part of the regenerative process as complete genetic ablation of reactive astrogliosis resulted in insufficient axonal regrowth (Faulkner et al., 2004; Anderson et al., 2016; Hara et al., 2017). Furthermore, networks of intermingled astrocytic protrusions serve as physical guidance structures for growing axons around spinal lesion sites (Silver et al., 1982; Fouad et al., 2005; Williams et al., 2015). Given these beneficial effects, astrocytes have been explored for restoration of spinal tissue integrity to mediate functional recovery (Chu et al., 2014). In line with the role of astrocytes in axonal guidance (Silver et al., 1982) and synapse formation (Baldwin and Eroglu, 2017; Liddelow and Barres, 2017) during development, it has been shown that immature (embryonic or neonatal) astrocytes provide greater regenerative bridging potential than mature astrocytes in the injured brain in both mice and rats (Smith and Silver, 1988; Smith and Miller, 1991; Filous et al., 2010). In a rat C3 fasciculus gracilis aspiration model, either oriented fetal E14 rat spinal cord tissue or astrocytes derived from E14 rat spinal cord were grafted (Bernstein and Goldberg, 1991). Over a 90 day period, the E14 derived astrocytes showed a significant increase in errors when crossing a horizontal ladder in comparison to the controls (aspiration-only), while the E14 rat spinal cord tissue graft showed a significant decrease in errors when crossing the ladder. This was found to be due to the astrocytic migration to the nucleus gracilis, where they protected neurons from denervation, increasing neuronal survival and networks only from astrocytes migrating out of the E14 spinal tissue but not from the E14 derived spinal cord astrocytes. Moreover, GFP labeled adult rat cortical astrocytes injected caudally (T11) 1 week after a T7-T8 full transection showed survival, integration and long distance migration to the lesion site and beyond 6 weeks post-transplantation (Pencalet et al., 2006).

Astrocytes are extremely heterogeneous and an adaptive cell population that vary morphologically and functionally, with phenotypes shifting with maturity, location, environmental cues and disease/injury-context (Zhang and Barres, 2010; Khakh and Sofroniew, 2015). For this reason, pre-cursors of the astrocytic and oligodendrocytic lineages (glial-restricted pre-cursors; GRPs) have been differentiated into homogenous subpopulations of astrocytes, GRP-derived astrocytes (GDA) differentiated with bone morphogenic protein 4 (BMP4, GDA^BMP4^) or with ciliary neurotrophic factor (CNTF, GDA^CNTF^). Firstly it should be noted, that after a T10 contusion in rats, transplantation of E13.5-derived GRPs alone retain their differentiation potential along the glial lineage, decrease reactive astrogliosis and CSPG expression levels as well as decrease axonal dieback of CST fibers from the lesion site and exhibit axonal growth cones (Hill et al., 2004). Injection of rat or human-derived GRPs or GDA^CNTF^ or GDA^BMP4^ into a rat C4/C5 dorsal column SCI model equally supported regeneration of ascending sensory tracts into the lesion site but not out (Haas et al., 2012; Haas and Fischer, 2013). Similarly, in a T10 moderate contusion in athymic rats human GRP and GDA^BMP4^ produced astrocytes at the lesion site that migrated out of the lesion and led to decreased cystic cavitation and reactive astrogliosis as well as increased sprouting. However, this did not lead to any significant changes in thermal and mechanical sensitivity or motor recovery (GridWalk) compared to controls (Jin et al., 2011). Several studies have been undertaken with GDA^BMP4^ showing increased growth of ascending sensory neurons of the dorsal columns into and through the lesion site, increased preservation of rubrospinal tracts and decreased misstep rates on the GridWalk (Davies et al., 2006; Fan et al., 2013; Wu et al., 2013), whereas GDA^CNTF^ has not shown any functional benefit or axonal regeneration but has led to allodynia as well as thermal and mechanical hyperalgesia (Davies et al., 2008, 2011).

Given that previous work with astrocytic grafting shows a lack of regenerative potential, astrocytes, themselves may be better suited as living scaffolds, linearly guiding axons in and out of biomaterials. Comparison of cultured astrocytes on either anisotropic poly(L-lactic acid) (PLLA) fibers or isotropic PLLA films revealed linear orientation of astrocytes to the anisotropic substrate, which provided a guidance matrix for the cultured astrocytes and dorsal root ganglia (DRG) neurons (Zuidema et al., 2015). When tethered self-aligning collagen gels aligned both the biomaterial with astrocytes this lead to significant increased growth of adult rat DRG in the aligned portions compared to the unaligned portions (East et al., 2010). Furthermore, alginate-based anisotropic capillary hydrogels lead to linear migration of astrocytes along capillary channels that are supportive of axonal outgrowth from neonatal rat entorhinal cortex and spinal cord slice cultures (Pawar et al., 2015b). Early studies already demonstrated that the implantation of fetal (E16 or E18) spinal cord astrocyte-seeded Millipore pennants (Category II) into a L5 dorsal root fiber crush model promoted substantial axonal growth into spinal gray matter tracts (Kliot et al., 1990). Additionally, implantation of neonatal rat cortical astrocytes situated in a Gelfoam matrix (Category II) or transplantation of astrocytes alone into a rat L3 dorsal lateral hemisection reduced host astrogliosis, scar formation and slightly increased density of neurofilament (NF) positive fibers when compared to controls (transplantation of Hanks buffered saline or implantation of empty Gelfoam) (Wang et al., 1995). While it is unclear which method has a greater beneficial effect, it was noted that the Gelfoam delayed the migration of the astrocytes from the lesion site and that cell-seeded Gelfoam appeared to be better integrated into the lesion site than Gelfoam alone. Fast blue labeled neonatal (P3) neocortical astrocytes embedded for retention in a collagen gel (modified Category I/II) were implanted into a 2 mm spanning T8 dorsal hemisection leading to increased neurofilament positive regenerating fibers within the implant, aligned along processes of the labeled astrocytes (Joosten et al., 2004). However, a minimal growth of biotinylated dextran amine (BDA) labeled CST fibers into the rostral edge of the gel scaffold was observed, although did not exit the lesion site. Limited improvements were observed in fine motor movements with the BBB subscore and Catwalk, analyzing hindlimb stride length and swing duration parameters, but not in the overall locomotor BBB score or Gridwalk analysis, when compared to collagen gel implantation alone. These changes may be due to the other fiber tracts not labeled in this study such as serotonergic fibers. To increase continuous tissue integrity across a large lesion site, Hoechst labeled P1 neonatal rat astrocytes were aligned on PLA/PLA-b-PEO matrices and implanted into a 2 mm T11/T12 dorsal hemisection with injection of astrocytes 1 mm both rostral and caudal of the lesion site (Category II and IV) (Deumens et al., 2006b). An increased BDA-labeled CST was observed up to the lesion site with astrocytes than controls (empty lesion site with media injected into the host parenchyma), however growth into the biomaterial was not observed likely due to the lack of astrocyte survival within the matrix. Naturally, no functional benefits were observed by the BBB locomotor score or stride length performed by Catwalk gait analysis.

Grafting of astrocytes, GRPs and GDAs into SCI lesions has shown their ability to reduce reactive astrogliosis, support neuronal survival and a minimal amount of axonal growth into but rarely beyond the lesion site. However, the regenerative potential of astrocytic grafting alone appears to be limited possibly due to their extensive migration away from the lesion. The combination of astrocytes with biomaterials delays their migration from the lesion site and enhances the host integration of the biomaterial by supplying directed growth of the axons along aligned astrocytes into the biomaterial. What remains to be examined is the use of GRPs or GDA^BMP4^, both of which had greater axonal support potential alone than neonatal astrocytes, in combination with biomaterials to not only enhance biomaterial integration through tissue continuity across the lesion but also possibly increase axonal growth. For combinatorial work with astrocytes (Table 3) likely other factors such as neurotrophic factors will need to be utilized to increase axonal regrowth beyond the lesion site.

**Table 3:** Astrocyte-seeded biomaterial SCI studies.

**SCI model**	**Biomaterial**	**Cell type**	**Outcome**	**References**
Rat L5 dorsal root fiber crush model	Millipore pennants (Category II)	Rat fetal (E16 or E18) spinal cord astrocytes	Promoted substantial axonal growth into spinal gray matter tracts	Kliot et al., 1990
Rat L3 dorsal lateral hemisection	Gelfoam matrix (Category II)	Neonatal rat cortical astrocytes	Reduced host astrogliosis, scar formation, and slightly increased density NF	Wang et al., 1995
Rat T8 dorsal hemisection	2 mm Collagen gel (modified Category I/II)	Neonatal rat (P3) neocortical astrocytes	Increased NF within biomaterial aligned along astrocytes, minimal CST growth into rostral end without exiting biomaterial, improvements in BBB sub-score and Catwalk stride length and swing duration	Joosten et al., 2004
Rat T11/T12 dorsal hemisection	2 mm PLA/PLA-b-PEO matrices (Category II and IV)	P1 neonatal rat astrocytes, 1 mm rostrocaudal injections	Increased CST growth up to the lesion site, poor astrocyte survival within matrix	Deumens et al., 2006b

### Olfactory ensheathing cells

Acquired from the olfactory bulb and mucosa, OECs represent an intermediary glial cell type between SCs and astrocytes (Granger et al., 2014). OECs express both the astrocytic marker GFAP (glial fibrillary acidic protein) and the SC marker p75-NTR (p75-neurotrophic factor receptor), however microarray profiling puts OECs in closer genetic proximity to SCs (Vincent et al., 2005). Similar to SCs, OECs have been shown to remyelinate injured axons (Li et al., 1997) and produce neurotrophic factors (Sasaki et al., 2006), although unlike SCs, OECs intermingle with host astrocytes to form supportive physical pathways presenting a growth inducing cellular and molecular substrate (Lakatos et al., 2000; Li et al., 2005). A review of OEC transplantation studies following SCI listed 41 studies showing beneficial effects, ranging from axonal regrowth, tissue sparring, angiogenesis, migration and remyelination, yet 13 studies showed no effects (Franssen et al., 2007). Several studies directly compared the transplantation of OECs or SCs and consistently observed benefits from SCs in axonal regeneration rather than OECs (Takami et al., 2002; Pearse et al., 2007). Furthermore, various OEC extraction protocols have led to varying purity which likely contributes to the observed high variability of effects of OECs in SCI studies (Ramon-Cueto and Nieto-Sampedro, 1992; Vincent et al., 2003; Rizek and Kawaja, 2006; Novikova et al., 2011).

In an attempt to further bridge nerve injuries, biomaterials have been combined with OECs to support axonal growth. OECs show a higher compatibility in terms of attachment and proliferation as well as nerve outgrowth on different biomaterials such as PLGA (Li et al., 2010), collagen (Wang et al., 2006), alginate and Matrigel scaffolds (Novikova et al., 2006) when combined with components of the ECM in a peripheral nerve injury model or *in vitro*. In rats a 2 mm long T11/T12 dorsal hemisection was filled with aligned OEC/ONF (olfactory nerve fibroblasts) -poly(D,L)-lactide biomatrix bridges (Category II) accompanied by Hoescht-labeled OEC/ONF injections surrounding the lesion site (1 mm rostral and caudal, Category IV), which led to migration of these cells up to the biomatrix but not within nor did the cells seeded within the biomatrix survive well, possibly due to the degradation of the biomatrix (Deumens et al., 2006a). All the same, this cell-seeded biomaterial implantation led to increased axonal growth into the lesion site than biomaterial alone, excluding CST growth, coupled with minimally increased gait parameters of stride length and swing speed (CatWalk) but no increased locomotor recovery (BBB). While the cause of this improvement was not specifically examined it was hypothesized to be due to other descending or ascending axonal tracts than the CST or the formation of new intraspinal relays. The use of OEC-seeded collagen-based multi-channel 3D matrices (Category II) in a 2 mm spanning T13 unilateral hemisection in rats showed no improvement on motor function (CatWalk) or alleviation of allodynia (von Frey hair filament test), furthermore axonal regrowth or scaffold integration was not examined (Deumens et al., 2013). Most recently, it was found that OEC-seeded PLGA scaffolds (Category II) in a 2 mm T9/T10 complete transection rat SCI model increased motor recovery (BBB score of 9 vs. 6 of controls and more successful crossings of an inclined plane) which correlated with increased axonal preservation and decreased astrogliosis reflecting neuroprotection as the underlying mechanism (Wang et al., 2017). As previously mentioned, the combination of OEC injection surrounding a PAN/PVC SC-seeded Matrigel implant in a full thoracic transection led to long distance growth both of ascending and descending axonal tracts into and through the lesion site (Ramon-Cueto et al., 1998; Fouad et al., 2005).

These studies (Table 4) show that OEC when injected surrounding the biomaterial help with growth of axons up to the lesion site, however they do not appear to migrate out from or into the biomaterial to increase tissue continuity, decrease cavitation surrounding the biomaterial or survive well when seeded in a biomaterial (this may have been due to biomaterial integrity, which should be re-examined), therefore currently at this time, OECs alone may not be the best candidate for combination with biomaterials.

**Table 4:** OEC-seeded biomaterial SCI studies.

**SCI model**	**Biomaterial**	**Cell type**	**Outcome**	**References**
Rat T11/T12 dorsal hemisection	2 mm poly(D,L)-lactide biomatrix (Category II and IV)	OEC/ONF within and 1 mm rostrocaudal injections	Migration of cells up to but not within biomatrix, poor cell survival within biomatrix, increased axonal growth excluding CST, increased stride length and swing speed	Deumens et al., 2006a
Rat T13 unilateral hemisection	2 mm collagen-based multi-channel 3D matrices (Category II)	OEC	No improvement in CatWalk gait analysis or alleviation of allodynia	Deumens et al., 2013
Rat T9/T10 complete transection	2 mm PLGA scaffolds (Category II)	OEC	Increased BBB score and crossing of inclined plane, increased axonal preservation, decreased astrogliosis	Wang et al., 2017

### Bone marrow mesenchymal stem cells

BMSCs are a widely used cell type for transplantation studies that can be easily isolated from a bone marrow aspiration and extensively expanded in culture, which makes autologous transplantation possible (Mendonca et al., 2014). BMSCs not only differentiate into a variety of mesodermal lines but also have been described to differentiate into microglia, oligodendrocytes and macrophages when transplanted into the spinal cord (Corti et al., 2002; Cizkova et al., 2006). However, the phenomenon of transdifferentation into neural cells has been challenged (Lu et al., 2004) and is likely not relevant for beneficial effects observed after SCI transplantation. More importantly for use in SCI, BMSCs can fill the lesion cavity and produce ECM components, thereby providing structural support for growing axons (Kim et al., 2013; Volpato et al., 2013). Furthermore, BMSCs display anti-inflammatory properties by producing immunoregulatory cytokines (interleukins and transforming growth factor-β) (Bartholomew et al., 2002; Noh et al., 2016) and interactions with host immune cells (Deans and Moseley, 2000; Zhang et al., 2016). In this context, transplantation of BMSCs diminished reactive astrogliosis and microglial activation (Abrams et al., 2009; Ruff et al., 2012). In addition to cytokines, BMSCs secrete permissive growth factors (Chen et al., 2002; Ohta et al., 2004; Kim et al., 2013; Ritfeld et al., 2015), although at relatively low levels. To enhance the role of BMSC paracrine secretion on axonal regeneration, genetically modified BMSCs have been used in various studies to deliver neurotrophic factors (Gong et al., 2015; Ritfeld et al., 2015; Zhu et al., 2015; Brock et al., 2016) in experimental SCI models. Although MSCs have been shown in a meta-analysis of relevant pre-clinical studies to increase the BBB score of thoracic SCI rats on average by 3.9 points the relevance of this increase has to be treated with caution due to the lack of a baseline from which locomotor activity is assessed (Oliveri et al., 2014).

While biomaterials fill the lesion site, they still require tissue continuity to allow for axonal growth into them. To this end, BMSC-seeded in porous but undirected 2-hydroxyethyl methacrylate (HEMA) or 2-hydroxypropyl methacrylamide (HPMA) scaffolds (Category II) implanted in a unilateral hemisection increased neurofilament positive axonal growth into the hydrogels (Sykova et al., 2006). Additionally, growth permissive BDNF expressing BMSCs were seeded into multi-component fiber bundled agarose scaffolds (Category II) after a 2 mm long T3 complete transection which led to increased growth of both descending (raphespinal and reticulospinal tracts) as well as ascending sensory fibers into the lesion site, far greater than GFP expressing BMSC-seeded biomaterials (Gao et al., 2013). To confirm this observation, BMSCs expressing BDNF seeded in a 2 mm alginate-based anisotropic capillary hydrogel (Category II) in a rat C5 unilateral hemisection enhanced directed axonal regrowth in comparison to hydrogels seeded with BMSCs alone (Gunther et al., 2015a,b). Both studies observed a significant increase of BDNF-driven axonal growth into and through the biomaterial but not exiting it nor did either study examine behavioral outcomes. By co-culture with NT3 overexpressing SCs in a 3D gelatin sponge scaffold, genetically modified BMSCs overexpressing Neurotrophic Receptor Tyrosine Kinase 3 (TrkC) differentiated into cells exhibiting neuronal features (neurofilament and post-synaptic density 95) (Category II), which were implanted into a rat 2 mm-wide T9-T10 transection SCI (Zeng et al., 2015). This treatment led to enhanced axonal growth throughout the biomaterial, synaptic association of these cells with mostly serotonergic neurons and some CST axons (electron microscopy), the upregulation of c-Fos in the grafted as well as host lumbar spinal cord cells in response to motor cortex stimulation and the improvement of locomotor function (BBB score of 8 for the cell-seeded biomaterial vs. 3 for biomaterial alone). The biomaterial alone vs. control SCI alone was not significantly different in axonal growth or functional parameters examined.

Overall, an added value of the combinatorial therapy with BMSCs and biomaterials is observable (Table 5) over biomaterials alone by increased ingrowth into scaffolds (Gao et al., 2013; Zeng et al., 2015), however it appears to not be sufficient enough growth for functionally relevant improvements alone without being coupled with other therapies such as neurotrophin overexpression or co-culture with other cell types.

**Table 5:** BMSC-seeded biomaterial SCI studies.

**SCI model**	**Biomaterial**	**Cell type**	**Outcome**	**References**
Rat unilateral hemisection	2-hydroxyethyl methacrylate (HEMA) or 2-hydroxypropyl methacrylamide (HPMA) scaffolds (Category II)	BMSC	Increased neurofilament positive axonal growth	Sykova et al., 2006
Rat T3 transection	2 mm multi-component fiber bundled agarose scaffolds (Category II)	BMSC-BDNF and BMSC-GFP	Increased growth of raphespinal, reticulospinal tracts and sensory fibers into but not exiting scaffold	Gao et al., 2013
Rat C5 unilateral hemisection	2 mm alginate-based anisotropic capillary hydrogel (Category II)	BMSC-BDNF and BMSC-GFP	Enhanced directed axonal regrowth into but not exiting hydrogels	Gunther et al., 2015a,b
Rat T9-T10 transection	2 mm 3D gelatin sponge scaffold (Category II)	co-culture SC-NT-3 and BMSC-TrkC	Enhanced axonal growth throughout the biomaterial, synaptic association of these cells with serotonergic neurons and some CST axons, upregulation of c-Fos in the grafted as well as host lumbar spinal cord cells and improvement of BBB score	Zeng et al., 2015

### Neural stem and progenitor cells

NSPCs can be generated from embryonic, fetal or adult CNS tissues and bear the unique feature of extensive self-renewal *in vivo* as well as differentiation into any desired neural cell type (Iwanami et al., 2005). NSPCs represent a promising and powerful tool to replace damaged tissue and bridge the lesion cavity by providing a cellular matrix, tissue replacement through targeted differentiation, neuroprotection and trophic support, (Assinck et al., 2017; Vismara et al., 2017). More recently, fetal spinal cell grafting after SCI is performed with growth factor trophic support and a fibrin matrix (Category I) to enhance cell survival and retention of the cells at the lesion site (Lu et al., 2012; Kadoya et al., 2016; Robinson and Lu, 2017). This encouraged the most extensive neuronal growth from NSPCs observed in a SCI (T3 full transection, CST and right quadrant lesions or C4 CST lesion) with resulting functional improvement in either hindlimb function (BBB score 6.5 vs. 1.5 BBB score for the lesion-only controls at 6 weeks post-grafting) or forelimb function (the staircase task with increased level reached and pellets eaten in the graft vs. lesion alone) or increased electrophysiology between grafted cells and CSTs through the creation of functional neuronal relays. Generally in the absence of growth factors and matrices, neural restricted progenitors (NRPs) and GRPs survive acute SCI grafting better than do multipotent neuroepithelial (NEP) stem cells, however delayed grafting of the fetal or embryonic NSPCs does allow for better survival and filling of the lesion site (Theele et al., 1996; Lepore et al., 2004; Iwanami et al., 2005; Lu et al., 2012, 2014a). It was found in mouse C4-CST lesions that grafted GRPs with NRPs or NPCs alone had surviving neurons and glia that filled the lesion site and supported CST regenerative growth, however grafted GRPs alone failed to do so (Kadoya et al., 2016). Moreover, it was found that adult NSPCs are incapable of filling the lesion cyst after transplantation into the injured spinal cord (Vroemen et al., 2003; Sandner et al., 2013). Another study has found that transplantation of NSPCs that differentiate primarily into astrocytes in response to SCI leads to the development of thermal and mechanical allodynia (Macias et al., 2006). Astrocytes do support axonal growth, however the type of growth they support may be determined by their dysregulation by the surrounding injury environment hence the development of astrocytic-dependent pain (Falnikar et al., 2015). Protection from direct contact with the lesion environment may provide NSPC-derived astrocytes with a different outcome (Cao et al., 2002).

Following SCI, the overall aim of seeding NSPCs into biomaterial scaffolds is to increase CNS regeneration by (1) improving the survival and potential differentiation of grafted NPSCs into mature cells to preserve tissue integrity by serving as a supportive matrix and (2) decreasing the host response to the biomaterial implantation by reducing inflammation and fibroglial scarring (Reeves and Keirstead, 2012; Bellenchi et al., 2013; Matsui et al., 2014). NSPCs derived from the subventricular zone (SVZ) of adult rats were incorporated into a PDGF-A-conjugated hyaluronan and methyl cellulose-based hydrogel blend (HAMC) (modified Category III/IV) and grafted 9 days post-clip T2 compression injury 1 mm rostral and 1 mm caudal to the injury site (Mothe et al., 2013). Even though cell survival was increased 1 week post-grafting, hardly any cells survived after 8 weeks when transplanted alone or in combination with the hydrogel. Nevertheless, sparing of host oligodendrocytes and neurons was enhanced, positively affecting functional outcomes for fine motor (horizontal ladder) changes but not gross motor skills (BBB). Murine NSCs (clone C17.2) were either seeded on a 4 mm oriented porous PLGA scaffold (Category II) or transplanted alone in comparison to naïve PLGA scaffolds or no treatment controls in a rat T9/T10 lateral hemisection model (Teng et al., 2002). To avoid additional neuroprotective effects no immunosuppression was used (Guo et al., 2001). Implanted scaffolds increased cell survival over transplanted cells alone. The greatest tissue preservation (white matter sparing) was served by the cell-seeded scaffolds followed by the scaffold alone, cells alone and finally the no treatment group. In addition to increased NF positive axonal growth within the lesion site, the scaffolds with or without cells had increased sensorimotor cortex traced BDA fibers both rostral and caudal to the lesion site co-labeled with growth-associated protein 43 kDa (GAP-43, an axonal regenerating marker) rostral to the lesion site, which was not observed in the cell transplantation or no treatment control groups. Both tissue preservation and regenerating fibers are thought to contribute to the increased motor recovery seen in the cell-seeded scaffold group (BBB score 11 vs. BBB score 8 for scaffold alone, and BBB score ~6 for cells alone or no treatment controls at 70 days). Implanted in a rat 2 mm full T8/T9 transection, PLGA scaffolds (with 7 longitudinal channels, each with a diameter of 660 μm) were seeded with rat E14.5 NSCs (telencephalon/diencephalon) or adult rat SCs suspended in Matrigel (Category II) or no cells (Olson et al., 2009). Even though seeded PGLA scaffolds increased axonal fibers throughout the scaffold after 4 weeks it did not lead to increased motor recovery in such a severe lesion (BBB score ~1). An oriented PLGA scaffold filled with a macroporous 4-arm poly(ethylene glycol) (PEG) hydrogel and coated with Poly(L-lysine) (PLL) was seeded with endothelial cells and NSPCs (from the SVZ zone of P1 GFP rats) in a rat T9-T10 lateral hemisection (Category II) (Rauch et al., 2009). This yielded a several fold increase in functional blood vessels over groups with either cell type seeded alone, biomaterial alone or lesion alone, recreating the blood spinal barrier. At 8 weeks post-injury, this also led to differentiation of the seeded NSPCs as well as increased NF staining at the host/graft interface and lesion epicenter coupled with GAP-43 staining of regenerating axons not colocalizing with GFP transplanted cells but with host axons. In another study, 10 mm long laminin-coated chitosan channels were seeded with either adult brain or spinal NSPCs (Category II) in a T8 transection rat model which led to long-term survival (14 weeks), differentiation (astrocytes and oligodendrocytes), decreased cyst formation and increased tissue bridge formation compared to empty scaffold or no scaffold (Nomura et al., 2008). Not surprisingly given the lesion size, no change in functional improvement occurred nor did any BDA labeled CST axons enter the channel. Implantation of adult neurosphere NSPC-seeded un-coated chitosan channels (Category II) 3 weeks post-T8 clip compression SCI in rats revealed a 5-fold increase in cell survival compared to NSPC grafting alone, however no tissue bridging or functional change was observed at 9 weeks (BBB scores ranging between 9 and 11) (Bozkurt et al., 2010). Non-proliferating NSPCs differentiated ~50% into oligodendrocytes, with very few differentiating into astrocytes or neurons, and ~50% remaining undifferentiated in the NSPC-seeded chitosan channels. NSPC grafting alone had greater number of oligodendrocytes and fewer undifferentiated cells, however overall cell survival was less than those seeded in channels.

To further improve these beneficial effects, either neurotrophic factors were overexpressed or bioactive molecules were conjugated into scaffold backbones. In an oriented macroporous PLGA scaffold seeded with rat P1-P3 hippocampal NSCs infected with NT-3 or TrkC (co-culture), naïve NSCs or unseeded PLGA were implanted into a 2 mm T10 full transection (Category II) (Du et al., 2011). Immunohistology and electron microscopy (EM) confirm differentiation into mature neurons (MAP2) forming synapases (PSD95) in the co-culture-seeded biomaterial group. This treatment also specifically led to the preservation of neurons in the sensorimotor cortex, red nucleus (descending tracts) and Clarke's nuclei (ascending tracts) as well as a significant increase in NF positive staining rostral and caudal to the scaffold and in the epicenter. Moreover, at 8 weeks post-injury this increased the motor recovery from 1.5 BBB score in the PLGA unseeded group to 3.5 in the NSC + PLGA group and 8.5 in the co-culture NT-3/TrkC-NSC + PLGA group. A similar study published at the same time in a T10 full transection implanted 2 mm Gelfoam scaffolds seeded with co-cultured NT-3-SC and TrkC-NSC (Category II) compared to Gelfoam alone, with NSC, with LacZ-SC + LacZ-NSC, with NT-3-SC + NSCs (Wang et al., 2011). The combination of NT-3-SC + TrkC-NSC seeded Gelfoam led to increased motor improvements (BBB 7.6 vs. NSC Gelfoam 1.6 and SCI alone 0.5, 60 days after injury) along with increased cortical somatosensory evoked potentials and cortical motor evoked potentials. This treatment also led to increased neuronal differentiation (MAP2), increased cell survival of internal pyramidal layer, red nuclei (descending tracts) and Clarke's nucleus (ascending tracts), increased SC myelination (EM) and synapse formation (EM and pre/post-synapse markers). While it needs to be further examined, the study indicates synapse formation at the epicenter of the lesion between transplanted NSCs and regenerating host axons leading to the enhanced functional recovery. A human immortalized NSC line (F3) was seeded with the addition of NT-3 expression in poly(ε-caprolactone) (PCL) scaffolds (Category II) in rat lateral T7-T8 hemisection which led to increased differentiation in neurons and oligodendrocytes, white matter sparing, regenerative markers (GAP-43 and synaptotagmin) by ELISA and Western blot analysis caudal to the lesion, than the F3-PCL group (Hwang et al., 2011). This correlated with increased motor recovery (BBB score up to 15 for the F3-NT-3-PCL, 13 for the F3-PCL, 11 for PCL and 10 for lesion alone as well as significantly less errors on a GridWalk at 4 and 7 weeks, errors decreasing with each additional treatment). Moreover, the addition of Chondroitinase ABC reduced CSPGs and increased motor recovery of the F3-NT-3-PCL group, including motor evoked potentials. Another study was conducted using rat neonatal NPCs (telencephalons) embedded into a 4 mm porous collagen and collagen-cetuximab scaffolds conjugated with a neutralizing antibody of epidermal growth factor receptor (EGFR, to block downstream inhibitory Nogo receptor signaling) (Koprivica et al., 2005), reducing the microglial inflammatory response (Qu et al., 2012) and reactive astrogliosis (Li et al., 2011) as well as increasing neuronal over astrocytic differentiation (Ayuso-Sacido et al., 2010) in a rat T13-L2 lateral hemisection (Category II) (Li et al., 2013). Here, neuronal differentiation was increased, whereas astrocytic differentiation was decreased and modest functional improvement was observed (BBB score 6 from 2 of empty collagen scaffold control and increased angle on inclined plane between seeded scaffolds vs. empty scaffolds).

In move toward clinical application both canine and African green monkey models of SCI (a 5 mm T11 lateral hemisection and a 10 mm T9/T10 lateral hemisection, respectively) have been developed to test the use of coated and uncoated PLGA scaffolds seeded with human NSC lines expressing NT-3 or not (Kim et al., 2010; Pritchard et al., 2010). While these studies found grafting to be feasible more work needs to be done to understand the efficacy of the treatments.

The combination of biomaterial scaffolds with NSPCs (Table 6) clearly has an enhanced effect on cell survival and to a lesser extent on differentiation as well as decreased cyst formation and increased tissue preservation. Many studies observed increased tissue bridging and regenerating axonal growth, however this was not always coupled with increased functional improvement. Importantly, with the addition of other factors, such as growth factors and biomolecules within the biomaterial, there was a greater increase in axonal growth into the scaffold, along with mature neuronal synapse formation with host neurons and supposed neuronal relays leading to functional improvements. Here, the combination of biomaterials with NSPCs (and other treatments) demonstrates a greater effect than NSPC transplantation alone after a SCI. Interestingly, this effect appears to be greater than the combination of biomaterials with other cell types.

**Table 6:** NSPC-seeded biomaterial SCI studies.

**SCI model**	**Biomaterial**	**Cell type**	**Outcome**	**References**
Rat T3 transection	Fibrin matrix (Category I)	Rat E14 fetal spinal cells with cocktail of growth factors	Neuronal differentiation and growth of NSPCs, extensive axonal growth (serotonergic) into the matrix creating neuronal relays, increased BBB score and electrophysiology	Lu et al., 2012
Rat bilateral CST and right quadrant lesions	Fibrin matrix (Category I)	Rat E14 fetal spinal cells with cocktail of growth factors	Neuronal differentiation and growth of NSPCs, extensive CST growth into the matrix creating neuronal relays, the staircase task with increased level reached and pellets eaten	Kadoya et al., 2016
Rat T2 clip compression	PDGF-A-conjugated HAMC (modified Category III/IV)	Adult rat SVZ-derived NSPCs	Enhanced sparing of host oligodendrocytes and neurons, increased fine motor (horizontal ladder) changes but not gross motor skills (BBB)	Mothe et al., 2013
Rat T9/T10 lateral hemisection	4 mm oriented porous PLGA scaffold (Category II)	Murine NSCs (clone C17.2)	Increased cell survival, tissue preservation, increased NF axonal growth within lesion site, increased sensorimotor cortex traced BDA fibers rostral and caudal to graft coupled with GAP-43, increased BBB	Teng et al., 2002
Rat T8/T9 transection	2 mm PLGA scaffolds (with 7 longitudinal channels, each with a diameter of 660 μm) and Matrigel (Category II)	Rat E14.5 NSCs (telencephalon/diencephalon) or adult rat SCs	Increased axonal fibers throughout scaffold but no change in BBB score	Olson et al., 2009
Rat T9-T10 lateral hemisection	Oriented PLGA scaffold with macroporous 4-arm PEG hydrogel coated with PLL (Category II)	Endothelial cells and NSPCs (from the SVZ zone of P1 GFP rats)	Increase in functional blood vessels, differentiation of NSPCs, increased NF staining at host/graft interface and epicenter and regenerating axons	Rauch et al., 2009
Rat T8 transection	10 mm laminin-coated chitosan channels (Category II)	Adult rat brain or spinal NSPCs	14 week survival, differentiation (astrocytes and oligodendrocytes), decreased cyst formation and increased tissue bridge formation, no CST growth into scaffold nor functional improvement.	Nomura et al., 2008
Rat T8 clip compression	Un-coated chitosan channels (Category II)	Adult rat neurosphere NSPC	5-fold increase in cell survival but no tissue bridging or functional change at 9 weeks	Bozkurt et al., 2010
Rat T10 transection	2 mm oriented macroporous PLGA scaffold (Category II)	Rat P1-P3 hippocampal NSC-NT-3/NSC-TrkC (co-culture), naïve NSCs	Differentiation into mature neurons with synapse formation, preservation of neurons in the sensorimotor cortex, red nucleus and Clarke's nuclei, increased NF rostrocaudal and epicenter staining, increased BBB score	Du et al., 2011
Rat T10 transection	2 mm Gelfoam scaffolds (Category II)	Co-cultured NT-3-SC and TrkC-NSC, naïve NSCs	Increased BBB score, increased cortical somatosensory evoked potentials and cortical motor evoked potentials, increased neuronal differentiation, increased cell survival of internal pyramidal layer, red nuclei and Clarke's nucleus, increased SC myelination and synapse formation	Wang et al., 2011
Rat lateral T7-T8 hemisection	NT-3 expression PCL scaffolds (Category II)	Human immortalized NSC line (F3) + ChABCase	Differentiation into neurons and oligodendrocytes, white matter sparing, regenerative markers (GAP-43 and synaptotagmin), increased BBB score and MEP, decreased errors on the GridWalk	Hwang et al., 2011
Rat T13-L2 lateral hemisection	4 mm porous collagen scaffold conjugated with neutralizing antibody of EGFR (Category II)	Neonatal NPCs	Increased neuronal differentiation, increased in BBB score and angle on inclined plane	Li et al., 2013

It should be noted that while not covered in this review that both the embryonic stem (ES) cells and induced pluripotent stem cells (iPSCs) are viable cellular candidates for biomaterial-supported cell transplantation. Both ES cells and iPSCs are capable of becoming any cell type and with this ability comes the potential for undifferentiated proliferation and tumorigenesis (Assuncao-Silva et al., 2015), which may be further enhanced by combination with a biomaterial. While ethical concerns constrict the use of ES cells and find benefit in the iPSCs which can be made autologously from a patient's skin sample, there still remains the concern of viral expression to induce pluripotency as well as the time frame it takes from collection of the patient sample until the iPSC or differentiated iPSC is produced. In addition, neuronal differentiation of iPSCs is more complicated than ES cells (Hu et al., 2010). In comparison to the work done with NSPCs and biomaterials in animal models, work done combining ES cells or iPSCs with biomaterials is more limited at this time (Hatami et al., 2009; Lu et al., 2014b; McCreedy et al., 2014).

## Challenges facing cell-seeded biomaterial strategies

More single application treatments (SCs, OECs, BMSCs, biomaterial scaffolds) are slowly making their way into clinical trials (Mackay-Sim et al., 2008; Kumar et al., 2009; Karamouzian et al., 2012; Tabakow et al., 2013; Amr et al., 2014; Mendonca et al., 2014; Theodore et al., 2016; Xiao et al., 2016; Anderson et al., 2017), however combinatory treatments are still primarily pre-clinical. Currently the field of cell-seeded biomaterials is in its infancy and targeting proof of concept experiments. If an experimental combination moves from *in vitro* to *in vivo* and shows substantial axonal regeneration linked to functional recovery then we can start addressing questions of clinical relevance. For example, many of the studies presented here were drastic hemisection or full transection injury models vs. the human relevant contusion/compression models (Devivo et al., 2002). In addition, many are thoracic lesions vs. the more common cervical lesions observed in SCI patients (Singh et al., 2014). In this thread, most studies work on acute or subacute SCI, as chronic SCI is time consuming and challenging for a treatment that has not already shown some promise in the acute phase. However, patients recruited for such clinical trials would likely be stable chronic patients who are no longer spontaneously recovering following the reduction of inflammation or other secondary damage, thus the obstacles faced in their lesion microenvironment would be different from that of the acute or subacute phase and must be taken into consideration.

In regards to cell transplantation in humans, autologous cells would be ideal, otherwise allogenic samples from other individuals may require long-term immunosuppression which will create other obstacles that need to be overcome. For some autologous cell types, longer periods of culturing are required to improve purity, quantity or differentiation which make immediate transplantation impossible in sub-acute SCI where much of the current preclinical work has been conducted (Mertens et al., 2016; Vismara et al., 2017).

Great progress has been made in both the fields of tissue engineering (cell purification procedures, autologous cell culture, culture methods increasing viability and genetic modifications) as well as biomaterial sciences (stability, combatibility, purity, self-assembling scaffolds, consistent capillary scaffolds, and biomolecule delivery), creating solutions (linear directed growth, increased viability, increased tissue stablity, decreased immune reactions) that were non-existent previously, allowing for new multifaceted approaches. Given these advances it may be of importance to revisit many combinations that were presented here but not performed under ideal conditions. For example, cell survival on some scaffolds was poor, likely due to the degradation of the scaffold during the study. Such work can now be repeated with more stable scaffolds and with additional coating of the scaffold with extracellular matrix proteins to increase survival and attachement (Hou et al., 2005; Tian et al., 2005). In addition, experiments comparing the combination of a single biomaterial with various cell types in a given SCI model would be beneficial in contrasting the effectiveness of each combinatorial therapy. For example, PGLA scaffolds seeded with NSPCs or SCs (Category II) were compared to empty scaffolds in a 2 mm full transection T8-T9 model, clearly showing that cell-seeded scaffolds increased axonal regeneration over empty control scaffolds and while not significant SC-seeded scaffolds trended to greater axonal regrowth than NSPC-seeded scaffolds (Olson et al., 2009). The current use of transgenic lines provides easy visual tracking of grafted cells allowing for a better understanding of cell survival, cell migration, differentiation, tissue bridging, synaptic connections and neuronal relays forming. For future studies it is of great importance that relevant functional assays be performed in combination with histological work along with proper controls showing the observed improvement can be directly linked to the observed host-graft integration or axonal regeneration, for example through a re-transection or ablation study.

## Perspectives of cell-seeded biomaterial strategies

The combination of cell transplantation and biomaterial scaffold implantation provides a promising tissue engineering strategy for SCI treatment that addresses the replacement of lost neural tissue and the support of axonal regeneration to achieve functional recovery. The poor survival rate of cells transplanted into the harsh post-SCI environment challenges their ability to fill and bridge the spinal lesion cavity or even provide physical and/or trophic support for axonal regrowth. In this context, biomaterial scaffolds provide a physical matrix for cell attachment, proliferation and differentiation that is protected from the harsh lesion microenvironment (Novikova et al., 2006; Führmann et al., 2017). In addition in such combinatorial approaches, the grafted cells aid scaffold integration into the host spinal environment by forming tissue bridges enticing axonal growth into and through the scaffold, recreating the lost neural tissue. While it is difficult to compare different SCI lesion models and severities, species, cell types and biomaterial scaffolds used, many studies indicate that cell-seeded biomaterial scaffolds lead to greater axonal regrowth and sometimes better functional outcomes than biomaterial scaffolds alone (Wang et al., 1995, 2011, 2017; Teng et al., 2002; Joosten et al., 2004; Deumens et al., 2006a; Nomura et al., 2008; Olson et al., 2009; Rauch et al., 2009; Du et al., 2011; Hwang et al., 2011; Gao et al., 2013; Li et al., 2013; Zeng et al., 2015). Additionaly, from a few studies presented here there is a strong indication that biomaterial-supported cell transplantation is greater than cell transplantation alone (Teng et al., 2002; Rauch et al., 2009; Bozkurt et al., 2010), unfortunately this is not a direct comparison that is often performed. Biomaterial-supported cell transplantation reduces tissue loss, inflammation and reactive astrogliosis, increases tissue integrity and bridging of the lesion site which has led to increased axonal growth across the lesion as well as increased functional improvements. Combining various cell types and growth factors to increase tissue bridging and integration of the biomaterial along with increased support of axonal regeneration not only into the biomaterial but also re-entry and long distance growth into the host parenchyma would likely substanstially promote functional improvements. Taken together thus far the work in biomaterial-supported cell transplantation strongly encourages a path forward toward combinatorial treatment of SCI.

## Author contributions

SL, TS, and RP: Researched, wrote, and edited the manuscript. NW: Edited the manuscript and provided expert feedback.

### Conflict of interest statement

The authors declare that the research was conducted in the absence of any commercial or financial relationships that could be construed as a potential conflict of interest.
